# Understanding the Mechanisms of Resistance to CAR T-Cell Therapy in Malignancies

**DOI:** 10.3389/fonc.2019.01237

**Published:** 2019-11-21

**Authors:** Jiali Cheng, Lei Zhao, Yuanyuan Zhang, Yun Qin, Yuqi Guan, Tong Zhang, Chaohong Liu, Jianfeng Zhou

**Affiliations:** ^1^Department of Hematology, Tongji Hospital, Tongji Medical College, Huazhong University of Science & Technology, Wuhan, China; ^2^Department of Microbiology, School of Basic Medicine, Tongji Medical College, Huazhong University of Science & Technology, Wuhan, China

**Keywords:** CAR T-cell therapy, resistance mechanism, T-cell defect, tumor factor, tumor microenvironment

## Abstract

Taking advantage of the immune system to exert an antitumor effect is currently a novel approach in cancer therapy. Adoptive transfer of T cells engineered to express chimeric antigen receptors (CARs) targeting a desired antigen has shown extraordinary antitumor activity, especially in refractory and relapsed B-cell malignancies. The most representative in this respect, as well as the most successful example, is CD19 CAR T-cell therapy in B-cell acute lymphoblastic leukemia (B-ALL). However, with the widespread use of CAR T-cell therapy, problems of resistance and relapse are starting to be considered. This review provides a comprehensive picture of the mechanisms of resistance to CAR T-cell therapy from three aspects, namely, CAR T-cell factors, tumor factors, and tumor microenvironment factors, offering insights for improving CAR T-cell therapy.

## Introduction

With changes in lifestyles and environments, the incidence of tumors, especially malignant tumors, has increased, threatening human health and society. In the centuries of battle against tumors, treatment strategies have evolved from surgery, radiotherapy, and chemotherapy to immunotherapy, which is more efficient and precise. Currently, immunotherapy includes antibodies, vaccines, immune checkpoint inhibitors, and adoptive cell transfer (ACT), such as T-cell receptor (TCR)-expressing T-cell infusion and chimeric antigen receptor (CAR)-expressing T-cell infusion (1–4). A common characteristic of active immunotherapy is that it utilizes the patient's own immune system to attack tumor cells. Among the available immunotherapies, the most encouraged is CAR T-cell therapy, which involves the genetic engineering of a patient's own T cells to kill tumor cells; it was first proposed and administered by the Israeli immunologist Zelig Eshlar in 1993 but has undergone a long and tortuous journey (5, 6). CAR T cells, a type of genetically engineered peripheral T cell, have a special antigen receptor whose extracellular single-chain variable fragment (scFv) can directly recognize a specific antigen independent of the major histocompatibility complex (MHC), an intracellular CD3ζ domain that conveys the T-cell activation signal, and a CD28 or 4-1BB domain that provides a costimulatory signal to facilitate the proliferation of CAR T cells and enable them to persistently attack tumor cells (7). First-generation CAR T cells contain an antigen-recognition domain and a CD3ζ domain; thus, although these cells can be activated, they are unable to proliferate (8). Second-generation CAR T cells introduce a costimulatory signal that enables T cells to proliferate after activation, making them a living drug *in vivo* (9).

Thus far, second-generation CAR T cells have been widely used in hematological malignancies, including B-cell acute lymphoblastic leukemia (B-ALL), B-cell Non-Hodgkin lymphoma (B-NHL), B-cell chronic lymphoblastic leukemia (B-CLL), and multiple myeloma (MM) (10–14), and have shown significant efficacy, as summarized in Table 1. The U.S. Food and Drug Administration (FDA) has approved anti-CD19 CAR T-cell therapy for patients with relapsed/refractory B-ALL and diffuse large B-cell lymphoma (DLBCL). Despite the impressive remission rates, some patients still relapse or are resistant to CAR T-cell therapy (15). Thus, when understanding the extraordinary efficacy, it is important for us to focus on unresponsive and relapsed cases to improve CAR T-cell therapy and facilitate the treatment of tumors. This article briefly reviews the efficacy and toxicity of CAR T-cell therapy, comprehensively analyzes the possible mechanisms of resistance to this therapy, and proposes possible solutions.

**Table 1:** Efficacy of CAR T-cell therapy in B-cell malignancies.

**Disease**	**Reference**	**CR/all cases**	**Follow-up**	**Long-term efficacy**
B-ALL (adult)	NCT01044069	44/53	Median: 29 m	median OS: 12.9 m
B-ALL (pediatric and young adult)	NCT02435849	61/75	12 m	OS rate: 76%
B-CLL	NCT01865617	4/19	24 m	median PFS: 8.5 m
DLBCL	NCT02348216	59/101	Median: 27.1 m	median PFS: 5.9 m
DLBCL	NCT02445248	37/93	Median:14 m	median OS: 12m
DLBCL	NCT02631044	33/73	Median: 8 m	DOR (CR; median): NR
MM (anti-BCMA)	NCT02215967	1/12^*^ (2 VGPR)	6 m	1 VGPR after 6 m
MM (anti-BCMA)	NCT02658929	15/33	Meidan:11.3	median DOR: 10.9

OS, overall survival; PFS, progression-free survival; m, months;

* *stringent complete response (CR); DOR, duration of response; NR, not reached*.

## Factors Related to the Efficacy and Toxicity of CAR T-cell Therapy

As summarized in Table 1, CAR T-cell therapy has shown impressive efficacy in B-cell malignancies, with a complete response (CR) rate of 81–90% for B-ALL and of approximately 50% for B-NHL (15–19). The cellular kinetics of CAR T cells and tumor burden are two important factors affecting the efficacy of CAR T-cell therapy in B-ALL (15, 20). AUC0-28d (area under the curve from the beginning to 28 days post-infusion), representing the expansion of CAR T cells, is a valuable parameter for predicting response: the larger the AUC0-28d, the better the response (21). The persistence of genetically engineered T cells is also an important factor affecting the prognosis of hematological malignancies. Integrated CD19 CAR transgene sequences can remain detectable over several years after infusion in peripheral blood, indicating that CAR T cells are able to survive in the body over a long period of time. Therefore, persistent remission after therapy is promising (21, 22). In addition, tumor burden is another important factor responsible for the prognosis of B-ALL patients. Heavy tumor burden, defined as 5% or more bone marrow blasts or extramedullary disease at the time of CAR T-cell infusion, is associated with a relatively higher chance of minimal residual disease (MRD)-positive remission (23). Research has indicated that the probability of relapse of a patient with CAR T-cell-induced MRD-positive CR is 100% (9 cases) and the probability of relapse of a patient with MRD-negative CR is 50% (16/32) (15). It is speculated that the ratio of AUC0-28d to tumor burden is a good indicator of the status of MRD and therefore the long-term prognosis of patients. Apart from the cellular kinetics of CAR T cells and tumor burden, the density of the targeted antigen, the tumor microenvironment, the status of donor T cells and the characteristic of disease, which can impact the recognition or cytotoxicity of CAR T cells, all have an impact on response to CAR T-cell therapy in B-cell malignancies (24).

The toxicity of CAR T-cell therapy mainly comprises the on-target effect, cytokine release syndrome (CRS), and neurologic toxicity. For anti-CD19 CAR T-cell therapy, B cell aplasia is a predictable on-target side-effect that impairs the humoral immunity and makes patients more susceptible to viral infections. Patients receiving CAR T-cell therapy may develop symptoms that differ from fever to hypotension, hypoxemia, and even multiple organ failure, usually with elevated cytokines like IL-6 and ferritin in serum, which is what is known as CRS. Park and collaborators reported that 45 out of 53 patients developed CRS during CAR T-cell therapy, 14 patients with grade 3 or higher CRS (15). Most cases of CRS can be managed through supportive care, tocilizumab, or corticosteroids. It is reported that the severity of CRS may be related to the tumor burden and the expansion of CAR T cells rather than the infusion dose of CAR T cells (23, 25). How CRS develops is still under research. Recently, a study by Li et al. suggested that TNF-α released by activated lymphocyte is key to inducing IL-6 and IL-1β secretion by monocytes and macrophages in the treatment of anti-HER2/CD3 bispecific antibody, which possibly exists in CAR T-cell therapy (26). Moreover, CAR T-cell therapy may result in neurotoxicity, manifested as cognitive defects, seizures, cerebral edema, etc. The mechanism of CAR T-cell therapy-related neurotoxicity also remains unclear. A study suggested that it may be due to endothelial damage and increased blood-brain barrier permeability (27).

In summary, AUC0-28d and tumor burden together with CAR T-cell persistence in peripheral blood affect the prognosis of patients. Most of the toxicities of CAR T-cell therapy are controllable, but the mechanisms of CRS and neurotoxicity remains to be further elucidated.

## Resistance to CAR T-cell Therapy

**Figure 1:**
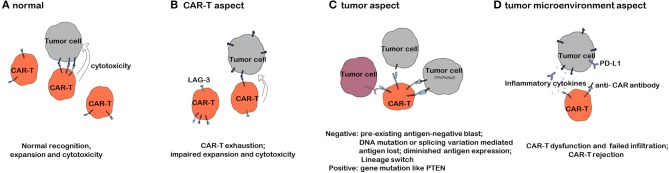
Mechanisms of resistance to CAR T-cell therapy. **(A)** CAR T cells exerting an antitumor effect under normal circumstances. **(B)** Mechanisms of resistance to CAR T-cell therapy with respect to CAR T cells. **(C)** Mechanisms of resistance to CAR T-cell therapy with respect to tumor cells. **(D)** Mechanisms of resistance to CAR T-cell therapy with respect to the tumor microenvironment.

Despite the impressive efficacy of CAR T-cell therapy in refractory/relapsed B-cell malignancies, the problem of relapse has gradually come to light with prolonged follow-up periods. According to data from different clinical trials, the relapse rate varies from 21 to 45% in B-ALL and increases with the follow-up time (23, 28, 29). We will discuss the mechanisms of resistance to CAR T-cell therapy, as shown in Figure 1, based on three aspects.

### T-Cell Factors

The response to tumor immunotherapy largely depends on the status of the immune function, suggesting that any defect in the immune system potentially attenuates the prognosis of patients. As shown in Table 1, the efficacy of CAR T-cell therapy in chronic lymphoblastic leukemia (CLL) is much worse than that in B-ALL, and this effect is proposed to be related to innate T-cell defects in CLL patients (13). In a phase I clinical trial of B-ALL (NCT01044069), 2 of 67 patients failed to produce CAR T cells, and in another clinical trial of DLBCL (NCT02445248), 12 of 165 patients experienced the same problem, suggesting that T cell defects have an impact on CAR T-cell production (15, 30). Expansion, persistence, and tumor cytotoxicity are the three main characteristics of CAR T cells that influence treatment efficacy. T cells from a cancer patient can often be deficient in their intrinsic cytotoxicity (31). CAR T cells derived from these cancer patients will thus have diminished cytotoxicity, resulting in a relatively poor prognosis. Transcriptome analysis has revealed that enhanced expression levels of key regulators of late memory/effector T-cell differentiation and aerobic glycolysis are associated with poor response to CAR T-cell therapy (32). Inhibition of glycolysis with 2-deoxy-D-glucose can facilitate the differentiation of central memory CAR T cells. Furthermore, studies indicate that the activation of interleukin (IL)-6/signal transducer and activator of transcription (STAT)-3 signaling pathways promotes central memory T-cell differentiation, which may play an important role in regulating the proliferation of CAR T cells (33). These findings suggest that the IL-6/STAT3 signaling pathway and glycometabolism can modulate the proliferation-dependent expansion of CAR T cells by regulating CAR T-cell differentiation. Furthermore, the constitution of the T-cell pool is constantly changing with age, shifting from undifferentiated naïve T cells to differentiated effector/memory T cells, which lack CD28 expression and have decreased proliferation ability when stimulated by antigens (34, 35). Whether the condition of the T-cell pool can influence the proliferation of CAR T cells has not been reported. In addition to impaired cytotoxicity and expansion, CAR T-cell exhaustion can lead to the failure of CAR T-cell therapy. T-cell exhaustion refers to a state of dysfunction characterized by a decrease in effectors and increased expression of inhibitory receptors, usually induced by chronic stimulation, such as in cancer (36, 37). B-cell recovery in peripheral blood is a marker of CD19 CAR T-cell dysfunction in the body. In pediatric B-ALL, B-cell recovery in peripheral blood within 3 months indicates a high risk of relapse, which may be due to CAR T-cell exhaustion (38). In DLBCL, a high percentage of LAG3^+^ T cells, a biomarker of T-cell exhaustion, is correlated with limited responses to CD19 CAR T-cell therapy (30). The mechanisms of CAR T-cell exhaustion are poorly understood. One study suggested that CARs on CAR T cells can spontaneously cluster in an antigen-independent manner, generating tonic CAR-CD3ζ signaling that can induce CAR T-cell exhaustion (39). Additionally, the endogenous TCR signal of CAR T cells in the presence of a specific antigen has been shown to induce T-cell exhaustion (40).

### Tumor Factors

Tumor factors are generally divided into two categories: relapse with positive target antigen expression and relapse with negative target antigen expression. Here, we discuss the two categories separately.

#### Targeted Antigen-Negative Relapse

Targeted antigen-negative relapse is one of the main reasons for resistance to CAR T-cell therapy and accounts for approximately 9–25% of cases of relapse in B-ALL according to several different clinical trials (15, 16, 20, 41). As recently reported at the 2018 ASH Meeting, CD19-negative and CD19-positive relapses occurred in 7/21 and 14/21 relapses, respectively, among DLBCL patients (42). The mechanisms of target antigen-negative relapse mainly involve four aspects: the preexistence of target antigen-negative tumor cells, diminished expression of target antigens, mutation-, splicing variation-, or lineage switching-mediated target antigen loss, and failure of presentation of target antigens. The efficacy of targeted therapy is thought to be tightly associated with the density of target antigens on the cell membrane. CD19, uniquely and broadly expressed in B-linage cells, is a favorable target antigen for CAR T-cell therapy (43). A research group from the Children's Hospital of Philadelphia (CHOP) investigated flow cytometric data from 628 cases of relapsed or refractory B-ALL and showed that before treatment, approximately 17% of cases had CD19-negative tumor cells (defined as the presence of more than 1% of negative cells), which may lead to relapse after anti-CD19 CAR T-cell therapy. Additionally, compared to healthy people, 7% of patients displayed diminished CD19 expression, and 24% of patients had low-normal CD19 expression (43). In addition to preexisting CD19^−^ or CD19^dim^ tumor cells, splicing variations and mutations partially accounted for CD19-negative relapse in CTL019 therapy (44, 45). Researchers in CHOP identified an SRSF3-involved alternative splicing of exon 2 of CD19 messenger RNA (mRNA) in CD19-negative relapsed B-ALL, which resulted in the loss of the targeted epitope in the membrane and consequent escape from the attack of anti-CD19 CAR T cells (44). Exon 5 and exon 6 deletion-mediated deficiency of the transmembrane domain of CD19 also induces CD19-negative relapse in response to anti-CD19 CAR T-cell administration. Mutations in exons 2–5, such as frameshift in exon 2, 3, or 4, insertion in exon 3, and nonsynonymous mutations in exon 4, lead to the loss of CD19 expression in the membrane and relapse post-CD19 CAR T-cell therapy (45). Lineage switching, referred to as conversions of leukemic cell lineage (46), can lead to CD19-negative relapse after CAR T-cell therapy. Research has shown that B-ALL cases with initial clearance of blasts post-infusion of CTL019 displayed CD19-negative relapse with a myeloid phenotype (47, 48). Experiments based on murine ALL models showed that in E2a:PBX1 B-ALL, the relapsed cells lost CD19 expression but expressed myeloid antigens post-CD19 CAR T-cell treatment, and the frequency was much lower in Eμ-RET B-ALL, suggesting that lineage switching-mediated CAR T-cell therapy resistance is related to genetic background (47). The exact mechanisms of lineage switching remain elusive, but there are two mainstream theories: drug-induced reprogramming of the original tumor stem cell and expansion of a different phenotypic clone of tumor cells during the process of targeted immunotherapy (49). Furthermore, deficient maturation and translocation of CD19 post-translation is a possible mechanism of CD19-negative resistance. A study identified three CD81 deficiency-related CD19-negative relapsed B-ALL cases post-blinatumomab therapy. Further investigation indicated a deficiency in CD81 expression resulting in failed formation of the CD19/CD21/CD81 coreceptor complex and hindered maturation and translocation of CD19 from the Golgi body to the cell membrane (50). Slightly different from CD19, the density of membrane CD22 differs considerably in normal situations, indicating that diminished CD22 site density is an important problem leading to antigen escape-mediated CAR T-cell therapy resistance. Additionally, approximately 22% of B-ALL patients are negative for CD22 according to a report from CHOP (43, 51).

#### Targeted Antigen-Positive Resistance

In clinical practice, target antigen-positive relapses can result from CAR T-cell defects. However, herein we will only discuss the tumor-related factors leading to target antigen-positive resistance. CAR T cells exert antitumor effects, which are dependent not only on the recognition of specific antigens but also on the induced apoptosis of tumor cells. Signals that induce apoptosis of tumor cells include tumor necrosis factor (TNF)-related apoptosis-inducing ligand (TRAIL), Fas ligand (FasL), and cytokines such as interferon (IFN)-γ (52–54). The mechanism of tumor antigen-positive resistance to CAR T-cell therapy underlies changes in tumor cell survival or apoptosis. Experiments have shown that even when the function of CD19 CAR T cells as measured using the secretion of type I cytokines is normal, a TRAIL inhibitor can suppress the cytotoxic effect of CAR T cells when the CAR T cells are cocultured with sensitive cells, indicating that a lack of TRAIL signaling in tumor cells can lead to tumor antigen-positive resistance to CAR T-cell therapy (55). To date, research on the relationship between tumor mutations and CAR T-cell therapy resistance remains limited. However, data from programmed cell death-1 (PD-1) therapy can provide some insights. In PD-1 immunotherapy of melanoma, PTEN deficiency leads to reduced tumor infiltration and decreased cytotoxicity of T cells, resulting in a poor response. Concurrent administration of PD-1/cytotoxic T-lymphocyte (CTL)-associated protein 4 (CTLA-4) inhibitor and PI3K-beta inhibitor can ameliorate the poor prognosis, indicating that PI3K-AKT over-activation is an important step in the process of antigen-positive resistance or relapse (56, 57). In addition, loss-of-function mutations in Janus kinase 1 (JAK1) or Janus kinase 2 (JAK2) can lead to resistance to PD-1 therapy in melanoma by blocking the IFN-γ signal (58). Whether the mechanisms of resistance to CAR T-cell therapy are similar to those to PD-1 therapy remains unknown.

In summary, target antigen-negative relapse mainly results from the preexistence or generation of targeted antigen-negative or targeted antigen-diminished tumor cells. As far as tumor-related factors are concerned, target antigen-positive resistance mainly results from tumor mutations such as those in PTEN and JAK1/JAK2, and this mechanism requires further investigation.

### Tumor Microenvironment

Studies on the role of the tumor microenvironment in CAR T-cell therapy are rare, which is likely due to the fact that CAR T-cell therapy is mainly used in hematological cancers such as B-ALL. The tumor microenvironment is mainly comprised of various cell types, including tumor-infiltrating immune cells, fibroblasts, and endothelial cells, and the extra-cellular cytokines, matrix, chemokine, etc., which modulate the development of tumor and the response to immunotherapy (59). The immunosuppressive tumor microenvironment in solid tumors is one of the most important factors impairing the efficacy of immunotherapy. The microenvironment interferes with the function and infiltration of immune effector cells. The phenomenon that tumor cells upregulate programmed cell death-ligand 1 (PD-L1) expression on the membrane to induce apoptosis in immune effector cells has been extensively studied in recent years (60–62). Recently, the Wei Gao group demonstrated that melanoma cells not only express PD-L1 but also release PD-L1 into the tumor microenvironment and blood circulation, leading to a poor response to cancer immunotherapy (63). As well as T-cell checkpoint blockage, hypoxia and glucose depletion result in lactic acid accumulation, consequently leading to low pH values in the tumor microenvironment, which suppresses the function of effector T cells, characterized by reduced IL-2 and IFN-γ secretion and lytic activity (64, 65). Additionally, specific components of the inflammatory tumor environment, for instance, Prostaglandin E2 (PGE2) produced by tumor cells in a mouse model, can affect the antitumor activity of T cells depending on IL-6, chemokine (C-X-C motif) ligand 1 (CXCL1), and granulocyte-colony stimulating factor (G-CSF) (66). Apart from cytotoxic T lymphocyte (CTL) dysfunction, cancer-associated fibroblasts (CAFs), myeloid-derived suppressor cells (MDSCs), and M2 subtypes of tumor-associated macrophages (TAMs) in the tumor microenvironment are reported to restrict infiltration of CTLs (67). On the other hand, the microenvironment trains tumor cells to disguise themselves to avoid recognition by immune cells. A clinical trial of Melan-A (MART-1)-targeted adoptive T-cell transfer therapy for metastatic melanoma revealed that TNF-α secreted by infiltrated CTLs induced dedifferentiation of melanoma cells to lose MART-1 and gp100 expression and acquire NGFR expression, leading to resistance to cancer immunotherapy (68).

An important concern in CAR T-cell therapy is the production of antibodies and CTLs against murine CAR scFv, which may result in CAR T-cell rejection. Most B-ALL patients who relapse after CD19 CAR T-cell therapy had no response to reinfusion of CD19 CAR T cells, even when CD19 expression is positive (31). In 2006, Kershaw reported that during anti-folic acid receptor CAR T-cell therapy for ovarian cancer, three of six cases displayed the presence of factors inhibiting CAR T-cell function in serum. After protein G administration, the inhibition was relieved (69). In 2013, Maus reported that during murine anti-mesothelin CAR T-cell therapy, after multiple reinfusion of CAR T cells, one patient developed an acute allergic reaction and died of cardiac arrest within a few minutes, which could be ascribed to the persistence of anti-CAR IgE in the body (70). According to the results reported by Turtle and collaborators, cytotoxic CD8^+^ T-cell responses to CAR T cells occurred in five CAR T-cell therapy-resistant patients, and the murine scFv FMC63 was identified to contain the immunogenic epitope (31). Together, these data suggest that anti-murine scFv CAR antibodies and CTLs are generated after CAR T-cell infusion, which can lead to CAR T-cell rejection. However, Mueller and collaborators reported that although 84.8% of patients generated anti-murine CAR antibodies after tisagenlecleucel treatment, it did not affect the efficacy of CAR T-cell therapy (22). Thus, the presence of an immune response to murine scFv and the consequence of this reaction remain unclear.

In summary, the tumor microenvironment is considered to be the bottleneck of CAR T-cell therapy in solid tumors, mainly due to its effect on defective CTL infiltration and dysfunction. Other important factors include anti-murine scFv antibodies and effector T cells, which may induce CAR T-cell rejection.

## Strategies to Overcome Resistance

We can improve CAR T-cell engineering to overcome the deficient cytotoxicity, expansion, and persistence of CAR T cells. To address an intrinsic deficiency in T cells, universal CAR T cells or haploidentical CAR T cells can be alternatively used (71). Generating universal CAR-T cells from allogeneic healthy donors required additional genetic modification to effectively abolish GVHD and/or CAR-T cell rejection. Universal CAR-T cell product offers a way to overcome the problem of quantitatively insufficient CAR-T cells from infants or highly treated patients who are profoundly lymphopenic owing to multiple previous chemotherapies. Qasim et al. demonstrated that two infants with relapsed refractory acute lymphocytic leukemia achieved molecular remission when treated with universal CAR-T cells (72). Besides, universal CAR T cells will make CAR T-cell therapy an off-the-shelf treatment, reduce the cost and time required to manipulate the patient's own T cells, and exclude the possible quality problems in T cells (73). To improve the proliferation of CAR T cells, modification of the costimulatory signal of CARs may also be an option. Currently, the most commonly used costimulatory signals of CARs involve 4-1BB and CD28. The 4-1BB signal induces moderate expansion and prolonged persistence of CAR T cells, but the CD28 signal induces robust expansion and relatively short persistence of CAR T cells (74), indicating that the costimulatory signal of CAR T cells can control the proliferation and persistence of these cells. Recently, third-generation CAR T-cells have been produced by incorporation of both the CD28 and 4-1BB co-stimulatory signals, expecting to obtain good anti-tumor potency and prolonged persistence at the same time. Preclinical data showed that third-generation CAR T-cells had balanced anti-tumor efficacy, improved persistence and decreased exhaustion compared with second-generation CAR T-cells (75). Clinical data confirmed the superior expansion and persistence of third-generation CAR T-cells (76). In a phase I/IIa clinical trial of third-generation CAR T-cells, 4 of 11 r/r B-cell lymphoma patients had initial CR, and another 3 patients achieved remission within 3 months. Two of 4 B-ALL patients had initial CR (77). More clinical data are required to inspect the efficacy of third-generation CAR T-cell therapy. Nevertheless, a better costimulatory signal remains to be discovered. Alternatively, we can infuse a specific composition of CAR T cells to improve proliferation and persistence. Studies have shown that an increased frequency of CD27^+^CD45RO^−^CD8^+^ CAR T cells, with a memory cell-like phenotype, can contribute to complete remission and prolonged event-free survival (78). Interestingly, biallelic inactivation of the gene Tet methylcytosine dioxygenase 2 (TET2) improves the persistence of CAR T cells, indicating the importance for identifying genes that determine the persistence of CAR T cells for the long-term prognosis in response to CAR T-cell therapy (79).

To circumvent antigen escape-mediated relapse, we can use CAR T cells targeting another antigen. A phase I trial reported that CD22 CAR T cells induced CR in 73% (11/15) of patients who had received CD19 CAR T-cell therapy and experienced a CD19-negative relapse or resistance, indicating that targeting another antigen may work in target antigen-negative relapse or resistance. However, 7 of 11 patients relapsed again with CD22^−^ or CD22^dim^ lymphoblasts, indicating that targeting another antigen is an effective but not a radical solution (51). Target escapes still happen against new agents. Another way to reduce target escape is to target multiple antigens at the same time or to sequentially infuse CAR T cells targeting different antigens in the beginning (80). To date, no data have been published to test the efficacy of these strategies. It is unknown whether the efficacy of sequential infusion of CD19 and CD22 CAR T cells is superior to that of infusion of CD19 CAR T cells alone at the beginning of therapy followed by infusion of CD22 CAR T cells after relapse. It should be noted that designing new target antigens is not always easy. Concerning the existence of CD19^dim^ or CD22^dim^ leukemic cells, researchers have tried to elevate the affinity between CAR and antigens to reduce the density of antigens required for CAR T-cell activation (81, 82). Nevertheless, the efficacy and safety of high-affinity CAR T cells remain to be evaluated. Immunogenic cell death is another potential strategy to overcome target antigen-negative relapse. Under certain conditions, cell death will activate the adaptive immune response (immunogenic cell death). The initiation of adaptive immune response mainly relies on two factors: adjuvants that can release a danger signal to trigger the immune response [it should be noted that some damage associated molecular patterns (DAMPs) suppress immune response instead of activating it (83)] and antigens that do not induce central and peripheral tolerance (84). Cancer cell death resulting in exposure of neoantigens to the immune system can, however, avoid its activation by limiting the danger signal (85). On the other hand, cell death induced by conditions such as chemotherapy or radiotherapy has been shown to release DAMPs, like CALR and HSP70, that promote the activation of the immune system (86, 87). Therefore, a combination of CAR T-cell therapy with radiotherapy, immune checkpoint inhibition, or vaccine may exert a synergistic anti-tumor effect because of the ICD-induced activation of the immune response. It remains unknown whether novel strategies, such as induction of tumor antigen expression, inhibition of targeted antigen loss, and targeting of a tumor marker at the DNA level, can help us win the battle against antigen-negative relapse in the future.

With regard to antigen-positive resistance, the main issue is that the cytotoxic signals emitted by CAR T cells fail to overcome the survival signals of tumor cells due to enhanced survival, proliferation, or cytotoxic signal shielding by tumor cells. Therefore, we can use target-specific drugs, such as PI3K-beta inhibitor, in combination with CAR T cells to regulate these signaling pathways and to counterbalance the abnormal proliferation and apoptosis signals in tumor cells. *In vitro* experiments have shown that the administration of the bcl-2 family apoptosis inhibitor ABT-737 can increase apoptosis in tumor cells induced by CAR T cells (88). Histone deacetylase inhibitors such as SAHA and LBH589 can also promote the sensitivity of resistant NHL cell lines toward CD19 CAR T cells by regulating apoptotic gene expression (55). Moreover, we can take advantage of the targeting ability of CAR T cells to accurately deliver drugs, thereby improving treatment efficacy and reducing side effects. In addition, hematopoietic stem cell transplantation (HSCT) is an alternative method, although there is still controversy as to whether HSCT after complete remission induced by CAR T-cell therapy benefits patients. Summers et al. reported that consolidative HSCT after CAR T-cell therapy in those ALL patients who have never received HSCT tends to improve the PFS, with a *p*-value of 0.059 (89). However, Park et al. reported that HSCT after CR induced by CAR T-cell therapy did not improve the PFS and OS, with a *p*-value of 0.64 for all CR patients and of 0.89 for MRD-negative CR patients (15). More clinical data are required to define whether HSCT is a beneficial consolidative treatment after CAR T-cell therapy.

The most attractive solution to overcome resistance due to the tumor microenvironment is to genetically engineer CAR T cells to secrete specific cytokines, such as IL-2 and IL-12. A phase I trial in 2005 reported that IL-12-secreting CAR T cells displayed stronger cytotoxicity and longer persistence during treatment in six cases of MUC16ecto+ ovarian cancer (NCT01457131). IL-12 is a proinflammatory factor that can activate the innate and adaptive immune systems to exert an antitumor effect and reduce the activity of regulatory T (Treg) cells and myeloid-derived immunosuppressive cells to counteract the immunosuppressive microenvironment (90). Based on the immune checkpoint theory, a more direct approach is to inactivate the immunosuppressive signal inside CAR T cells through gene-editing technology, to engineer CAR T cells to secrete PD-1 inhibitors, or to combine PD-1 blocking antibodies with CAR T cells (NCT02926833). It has been reported that knocking down *PDCD1*, the gene encoding PD-1, can increase the antitumor activity of CAR T cells (91). CAR T cells can also be engineered to secrete some enzymes or chemokines, such as heparanase, to promote the infiltration of immune effector cells into tumor, especially in solid tumors. For antibodies against murine CAR scFv, the application of humanized CAR T cells is the best solution.

## Concluding Remarks

The emergence of CAR T-cell therapy has altered the landscape of cancer immunotherapy, showing an impressive outcome in B-cell malignancies. Two CD19 CAR T-cell therapies have been approved for the treatment of B-ALL and DLBCL. However, resistance, both primary and acquired, to CAR T-cell therapy can still emerge. One of the most important goals of the field is to determine the signals triggered by CAR stimulation, which is fundamental for advancing CAR T-cell therapy. Immune escape of target antigen-negative tumor cells also occurs in CAR T-cell therapy, which could be managed by targeting another antigen. Nevertheless, resistance to the new target antigen can also occur in theory. This situation is similar to a race, i.e., if immune effector cells can find all tumor cells before they are masked, the tumor loses; otherwise, the treatment is unsuccessful. Additionally, the tumor microenvironment, a complicated and dynamic environment, can hamper the efficacy of CAR T-cell therapy, especially in solid tumors. Advances in gene-editing technology and cell culture technology may facilitate the efficacy of CAR T-cell therapy. Nonetheless, tumor cells are evolving, and, thus, mechanisms to radically avoid immune escape remain to be explored. There is still a long way for humans to go to defeat cancer. Finally, apart from the accessibility of technology, the heavy economic burden of CAR T-cell therapy has limited the use of the therapy for cancer. In the USA, the price of Tiga-Cel (“Kymriah”) is $475,000 and that Axi-Cel (“Yescarta”) is $350,000, which most countries in the world cannot afford. In all, CAR T-cell therapy represents one of the most effective and advanced treatments in B-cell malignancies, although it still faces some challenges, namely that defects in CAR T-cell, targeted antigen escape, tumor mutation, and the tumor microenvironment can result in resistance or relapse to CAR T-cell therapy. However, as the development of science and technology continues, CAR-based cellular immunotherapy will become more powerful.

## Author Contributions

JC wrote the initial draft. LZ revised the review. YZ, YQ, YG, TZ, and CL discussed the review and approved the final manuscript. JZ conducted the whole process. All authors checked and approved the final version.

### Conflict of Interest

The authors declare that the research was conducted in the absence of any commercial or financial relationships that could be construed as a potential conflict of interest.

## Abbreviations

ACT: adoptive cell transfer
AUC: area under the curve
B-NHL: Non-Hodgkin lymphoma
B-CLL: B-cell chronic lymphoblastic leukemia
B-ALL: B-cell acute lymphoblastic leukemia
CAR: chimeric antigen receptor
CD3: cluster of differentiation 3
CR: complete remission
CRS: cytokine release syndrome
CTLA-4: cytotoxic T-lymphocyte-associated protein 4
CTL: cytotoxic T lymphocyte
CAF: cancer-associated fibroblast
CXCL1: chemokine (C-X-C motif) ligand 1
Dim: diminished
DNA: deoxyribonucleic acid
DLBCL: diffused large B cell lymphoma
FDA: U.S. Food and Drug Administration
G-SCF: granulocyte-colony stimulating factor
HSCT: hematopoietic stem cell transplantation
IL-6: Interleukin 6
ICD: immunogenic cell death
IFN: interferon
JAK: janus kinase
LAG-3: Lymphocyte-activation gene 3
MHC: major histocompatibility complex
MM: multiple myeloma
mRNA: messenger RNA
MLL: Histone-lysine N-methyltransferase
MRD: minimal residual disease
MART-1: melan-A
MDSC: myeloid-derived suppressor cell
p53: cellular tumor antigen p53
PR, partial remission PI3K-AKT: phosphatidylinositide 3-kinases-Protein kinase B
PD-1: programmed cell death-1
PD-L1: programmed cell death-ligand 1
PGE2: prostaglandin E2
scFv: single-chain variable fragment
STAT3: transducer and activator of transcription 3
TCR: T cell receptor
TRAIL: tumor necrosis factor-related apoptosis-inducing ligand
TNF: tumor necrosis factor
Treg: regulatory T cell
TET2: Tet methylcytosine dioxygenase 2
TAM2: tumor-associated macrophage.
